# Mitochondrial-Targeted Antioxidant MitoQ Prevents* E. coli* Lipopolysaccharide-Induced Accumulation of Triacylglycerol and Lipid Droplets Biogenesis in Epithelial Cells

**DOI:** 10.1155/2018/5745790

**Published:** 2018-09-02

**Authors:** Ekaterina Fock, Vera Bachteeva, Elena Lavrova, Rimma Parnova

**Affiliations:** I. M. Sechenov Institute of Evolutionary Physiology and Biochemistry of the Russian Academy of Sciences, Saint-Petersburg, Russia

## Abstract

The effect of bacterial lipopolysaccharide (LPS) on eukaryotic cell could be accompanied by a significant metabolic shift that includes accumulation of triacylglycerol (TAG) in lipid droplets (LD), ubiquitous organelles associated with fatty acid storage, energy regulation and demonstrated tight spatial and functional connections with mitochondria. The impairment of mitochondrial activity under pathological stimuli has been shown to provoke TAG storage and LD biogenesis. However the potential mechanisms that link mitochondrial disturbances and TAG accumulation are not completely understood. We hypothesize that mitochondrial ROS (mROS) may play a role of a trigger leading to subsequent accumulation of intracellular TAG and LD in response to a bacterial stimulus. Using isolated epithelial cells from the frog urinary bladder, we showed that LPS decreased fatty acids oxidation, enhanced TAG deposition, and promoted LD formation. LPS treatment did not affect the mitochondrial membrane potential but increased cellular ROS production and led to impairment of mitochondrial function as revealed by decreased ATP production and a reduced maximal oxygen consumption rate (OCR) and OCR directed at ATP turnover. The mitochondrial-targeted antioxidant MitoQ at a dose of 25 nM did not prevent LPS-induced alterations in cellular respiration, but, in contrast to nonmitochondrial antioxidant *α*-tocopherol, reduced the effect of LPS on the generation of ROS, restored the LPS-induced decline of fatty acids oxidation, and prevented accumulation of TAG and LD biogenesis. The data obtained indicate the key signaling role of mROS in the lipid metabolic shift that occurs under the impact of a bacterial pathogen in epithelial cells.

## 1. Introduction

Bacterial lipopolysaccharide (LPS), the main membrane component of Gram-negative bacteria, is one of the most important pathogen-associated molecular patterns, which elicits the host innate immune response as well as inflammation. The effect of LPS on eukaryotic cells could be accompanied by a significant metabolic shift including accumulation of triacylglycerol (TAG) deposited in lipid droplets (LD), ubiquitous organelles that are associated with fatty acid storage, energy regulation, and control of bioactive lipid mediator production [1, 2]). Both* in vitro *and* in vivo,* LD-associated accumulation of TAG in response to LPS has been shown mainly in immune cells such as macrophages [3, 4], leukocytes [5], and microglia [6]. Systemic administration of LPS has been shown to cause an increase in the TAG content in the kidney, liver, and heart [7–10].

The effect of LPS on intracellular TAG accumulation has been evidenced to be based on multifaceted and highly cell-type specific pathways. Among them are the increase of CD36-mediated uptake of fatty acids and their incorporation into TAG [3, 4], the decrease of adipose triglyceride lipase- (ATGL-) mediated TAG lipolysis [4], the impairment of fatty acids oxidation (FAO), and downregulation of expression of the transcriptional factor PPAR*α* and its downstream genes that are required for FAO [7, 8, 11, 12]. However, these effects can be triggered by earlier step(s) in LPS signaling, initiating alterations in the expression and activity of proteins involved in cellular lipid metabolism. These steps are still poorly understood.

LD has tight spatial and functional connections with mitochondria, and impairment of mitochondrial activity provokes TAG storage and LD biogenesis [13–16]. In different cell types, challenge with LPS causes an increase in reactive oxygen species (ROS) generation, a decline in mitochondrial membrane potential (MPP) and respiratory complexes activity, and a decrease in the oxygen consumption rate (OCR) and ATP production [17–21]. Mitochondria, especially complexes I-III of the electron transport chain (ETC), are the predominant cellular source of ROS which are important for cellular signaling and are tightly regulated by the endogenous antioxidant scavenging system [22, 23]. The link between ETC disturbances, mitochondrial ROS (mROS) generation, and TAG accumulation was revealed from data that showed that antimycin, an inhibitor of respiratory complex III, whose effect may be coupled to mROS generation, causes a decrease of FAO and stimulates TAG accumulation [10, 13, 24]. The amount of LD has been shown to be increased in the glia of mitochondrial mutants with elevated level of ROS, and reduction of ROS prevents LD accumulation [16]. These data prompted us to suggest that mROS trigger subsequent accumulation of intracellular TAG and LD in response to a bacterial stimulus.

To clarify the involvement of mROS in the LPS-induced shift of lipid metabolism, in this study we used MitoQ, a ubiquinone derivative that is covalently attached to a lipophilic triphenylphosphonium cation. Such a structure and a high potential across the inner membrane of mitochondria allow MitoQ and other structurally similar compounds to be extremely highly concentrated in mitochondria matrix scavenging active radicals [25]. The protective effect of mitochondrial-targeted antioxidants against LPS-induced inflammation or even acute sepsis has been shown in different* in vivo* models [26, 27]. In* in vitro *experiments with LPS, mitochondrial-targeted antioxidants have been shown to prevent the increase of proinflammatory cytokine production in macrophages [28] and suppressed NF-kB and MAPKs activation in microglial cells [29]. However, the potential link between mROS generation and LD biogenesis in response to LPS has not been studied yet.

As a cellular model, we used epithelial cells from the frog urinary bladder mucosa (FUBEC,  *rog*  *rinary*  *ladder*  *pithelial*  *ells*). Epithelium of the urinary bladder forms a barrier to pathogen entry and is the first line of defense against penetrating microorganisms. For this reason, uroepithelia of different animal species possess an arsenal of tools for the innate immune defense, including the recognition of pathogen factors and TLR-triggered generation of a variety of inflammatory mediators [30–32]. As we reported previously, FUBEC express TLR4 and respond to LPS via a cascade of inflammatory signaling events leading to the increase of iNOS expression and PGE_2_ synthesis [30]. Importantly, as in other cells, FUBEC accumulate LD in response to exogenously added fatty acids demonstrating the existence of LD-biogenesis machinery [33].

In this study, we characterized the effects of LPS on the rate of fatty acid oxidation (FAO), TAG storage, and LD accumulation and analyzed LPS-induced alterations in mitochondrial function. With the use of the mitochondrial-targeted antioxidant MitoQ, we tried to demonstrate whether mROS play the trigger role in LPS-induced lipid metabolic shift.

## 2. Materials and Methods

### 2.1. Reagents

*E. coli *LPS (serotype 0127:B8), Leibovitz L15 medium, oligomycin, FCCP (carbonyl cyanide-p- trifluoromethoxyphenylhydrazone), rotenone, antimycin, myxothiazol, *α*-tocopherol, 3-(4,5-dimethylthiazol-2-yl)-2,5-diphenyltetrazolium bromide (MTT), 2,7-dichlorofluorescein-diacetate (DCF-DA), and lipid standards were from Sigma-Aldrich (St. Louis, MO, USA). Nile Red was from Invitrogen (Carlsbad, CA, USA). [9, 10- ^3^H(N)]-oleic acid was from Perkin Elmer (Boston, MA, USA). 5,5',6,6'-Tetrachloro-1,1',3,3'-tetraethylbenzimi-dazolylcarbocyanine iodide (JC-1) was purchased from Molecular Probes (Eugene, OR, USA). MitoQ was a kind gift from Dr. Michael Murphy (Cambridge, UK).

### 2.2. Animals

Male frogs* Rana temporaria L*., which originated from the wild population in the Northern European region of Russia, were kept for 2-4 weeks in a hemiaquatic bath at +5°C. All procedures using animals were performed in accordance with the European Communities Council Directive (24th November 1986; 86/609/EEC) and were approved by the local Institutional Animal Care and Use Committee.

### 2.3. Culturing of FUBEC

The experiments were carried out on frog urinary bladder epithelial cells isolated as described previously [34]. Cells were washed with sterile amphibian Ringer solution (ARS), containing 85 mM NaCl, 4 mM KCl, 17.5 mM NaHCO_3_, 0.8 mM KH_2_PO4, 2 mM glucose, 1.5 mM CaCl_2_, 0.8 mM MgCl_2_, and 40 *μ*g/ml gentamycin at pH 7.6, and they were then resuspended in Leibovitz L-15 medium diluted with ultrapure water for adaptation to frog osmolality (230 mOsmol/kg H_2_O), and finally they were supplemented with 40 *μ*g/ml gentamycin. Cells were incubated in 24-well plates at 23°C in a humid chamber (1.5−2 × 10^6^ cells/250 *μ*l per well).

### 2.4. Cell Viability Assay

Cells were seeded onto 96-well plates at a density of 1.5 x 10^5^ cells/well and were incubated for 21 h with different concentration of MitoQ. MTT-reagent (at a final concentration of 0.5 mg/ml) was added 3 h before the end of the incubation, and then cells were lysed by a mixture of isopropanol: HCl (100: 1). The colored product of the MTT reduction was scanned with a microplate reader at 570 nm using 620 nm as the reference wavelength. The results were expressed as a percentage of the optical density, with the control taken as 100%. Since MitoQ was dissolved in 96% ethanol, corresponding volumes of the solvent were added. The ethanol concentration was less than 0.5%.

### 2.5. Lipid Droplets Staining

The stock Nile Red solution in DMSO (1 mg/ml) was diluted* ex tempore* 1000-fold with L-15 medium. At the end of incubation with or without LPS, 100 *μ*l of the diluted Nile Red solution was added to 250 *μ*l of the cell suspension for 10 min. Then, the suspension was transported to a confocal camera and microphotographs were obtained with a Leica TCS SP5 MP microscope (*λ*_ex_ = 488 nm, *λ*_ex_ = 510 – 560 nm, dry objective x 40).

### 2.6. Oxygen Consumption Rate (OCR) Analysis

OCR (nmol per minute per 10^6^ cells) was measured using a polarographic oxygen Clark-type electrode (Econix-Expert Ltd, Russia) at 23°C under constant stirring. At the end of the appropriate incubation, 1.3 ml of the cell suspension containing 5-7 x 10^6^ cells was placed in a polarographic chamber. Respiration was allowed to stabilize before any additions. Then, oligomycin (the ATP-synthase inhibitor, 1.5 *μ*M) was added to estimate respiration independent of ATP synthesis. To evaluate the maximal capacity of ETC, the protonophore FCCP was titrated at different concentrations (0.75-1.25 *μ*M) until a maximal respiration level was reached. The respiration was then inhibited by adding 2 *μ*M rotenone, a complex I inhibitor, and 2 *μ*M antimycin and 1 *μ*M myxothiazol, complex III inhibitors. Finally, the addition of 2 mM KCN enabled the measurement of nonmitochondrial OCR. The basal OCR was calculated as the OCR before any additions minus the nonmitochondrial OCR. OCR_ATP_ was determined as the basal OCR minus OCR after oligomycin addition.

### 2.7. ROS Measurement

Cells were seeded onto 96-well plates at a density of 1.5-1.8 × 10^5^ cells per well and incubated for 2 h with or without MitoQ followed by exposure to LPS for 1 h. The fluorescent dye DCF-DA was added to the incubation medium, at a final concentration of 10 *μ*M, 20 min before the end of the incubation with LPS. The fluorescence of the reaction product of ROS with DCF-DA was estimated by a Fluoroscan FL (Thermo Fisher Scientific, Waltham, MA) at *λ*_em_ = 538 nm and *λ*_ex_ = 485 nm. The ROS content was expressed in arbitrary units.

### 2.8. Mitochondrial Membrane Potential Evaluation and ATP Assay

At the end of the appropriate incubation, cells were rinsed with ARS, resuspended in 50 *μ*l of the same solution, incubated with JC-1 at a concentration of 2.5 *μ*g/ml for 20 min in the dark, and analyzed on a Navios flow cytometer (Beckman Coulter) at FL1 (525 ± 40 nm) and FL2 (575 ± 30 nm), with *λ*_ex_ = 488 nm. The change in color from red to green was quantified and analyzed.

Cellular ATP production was evaluated by the luciferin-luciferase method using a commercial kit (Lumteck, Moscow) according to the manufacturer's protocol.

### 2.9. Lipid Extraction and Separation of Lipid Classes by TLC

FUBEC were incubated with or without LPS for 21 h in 24-well plates at a density of 1.5 x 10^6^ cells/well. At the end of the incubation, cells were harvested, washed, and subjected to lipid extraction by a chloroform/methanol (2:1) mixture. Lipid extracts were washed with 0.2 vol of a 0.75% KCl solution and centrifuged for 5 min at 250 g. The lower phase was evaporated until dry, and the sediment dissolved in a chloroform-methanol mixture (2:1, v/v) was applied to a chromatographic plate DC-Alufolien (Merck, Germany). Lipid classes were separated in a solvent system containing hexane-diethyl ether-acetic acid (33:11.3:1, v/v). The plates were sprayed with 20% H_2_SO_4_ in methanol and heated at 150°C. Lipid spots were identified with the use of corresponding standards. For quantitative analysis, the plates were scanned and the densities of the lipid spots were measured by ImageJ and Microsoft Office Excel. The absolute values of TAG were calculated based on the optical density of a known concentration of triolein solutions applied on the same plate.

### 2.10. Incorporation of [^3^H]-Oleic Acid into Lipids and Evaluation of FAO

Freshly isolated and washed cells (1.2 x 10^6^ in each sample) were incubated with 26 pmol of [9,10- ^3^H(N)]-oleic acid (specific activity of 45.5 Ci/mmol) for 1 h at 23°C. After that cells were washed with ARS, resuspended in culture medium, and incubated for 21 h with or without LPS. Where appropriate, before LPS, cells were incubated for 2 h with 25 nM MitoQ. At the end of the incubation, cells were centrifuged for 10 min at 100 g and the supernatant was gathered. The pellet was subjected to the lipid extraction procedure followed by separation of lipid classes by TLC as described above. The plate was developed in iodine vapor. After iodine evaporation, zones corresponding to lipid classes were cut out and their radioactivity was measured by an LKB 1209/1215 Rack-Beta counter.

For FAO evaluation, at the end of the incubation, aliquots of extracellular medium were mixed with 4 vol of chloroform-methanol (2:1). The oleic acid oxidation rate was evaluated by measurement of the radioactivity of the water phase. The scintillation count was normalized to 10^6^ cells.

### 2.11. Statistics

The results are presented as the means ± SE. Statistical analysis was performed with the help of Microsoft Office Excel and the statistical software package AtteStat, version 13.1. The Shapiro-Wilk test was used to check samples for normality. The statistical significance of differences was determined by Student's* t*-test for paired samples or one-way ANOVA where appropriate. Differences between tests and controls were considered statistically significant at* P* value <0.05.

## 3. Results

### 3.1. LPS-Stimulated Lipid Droplet Biogenesis and Intracellular Accumulation of TAG

**Figure 1:**
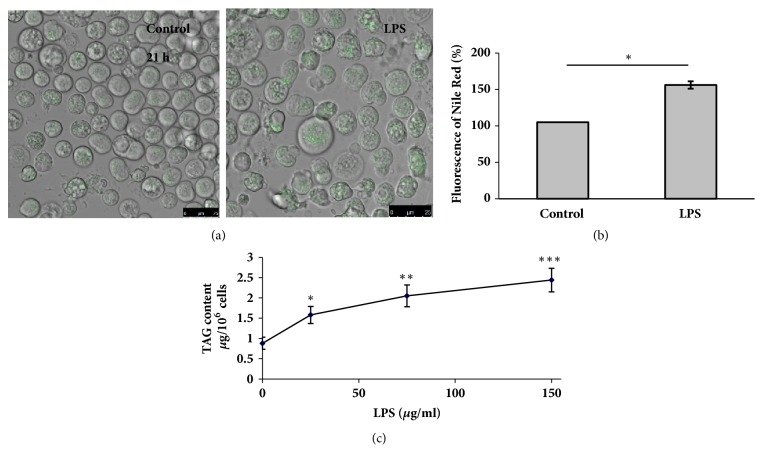
LPS stimulates lipid droplet biogenesis** (a, b) **and intracellular accumulation of TAG** (c)**.** (a, b)** FUBEC incubated with LPS (25 *μ*g/ml) or without it for 21 h were stained with Nile Red for 10 min. The suspension was then transferred to the confocal camera, and microphotographs were obtained with a Leica TCS SP5 MP microscope (*λ*_ex_ = 488 nm, *λ*_ex_ = 510 – 560 nm, dry objective x40). *∗* p<0.05 (n=4).** (c)** Dose dependency of LPS effect on the intracellular TAG content. Cells were incubated with or without LPS for 21 h, harvested, and subjected to lipid extraction and TLC. n=6 independent experiments. *∗* p<0.05; *∗∗* p< 0.01; *∗∗∗* p<0.001 versus control (the zero point on the x-axis).

The neutral lipid fluorescent dye Nile Red revealed the presence of LD at different quantities in virtually all control FUBEC, nonuniformly distributed in the cytoplasm, sometimes forming aggregates (Figure 1(a)). Cells treated for 21 h with LPS displayed a significantly elevated number and size of LD (Figures 1(a) and 1(b)). The increase of Nile Red fluorescence in the selected range of wavelengths may have been caused by augmentation of both of TAG and the cholesterol ester (CE) content. However, densitometric analysis of the thin-layer chromatograms of the total lipid extracts displayed a dose-dependent increase of the TAG content in the presence of LPS (Figure 1(c)), whereas CE deposition was not promoted (data are not shown).

### 3.2. LPS Inhibited FAO and TAG Breakdown

To understand the metabolic origin of TAG accumulation in response to LPS, we preincubated FUBEC with [^3^H]-oleic acid before challenge with LPS and then examined the incorporation of the radioactive label into the main lipid classes.

**Figure 2:**
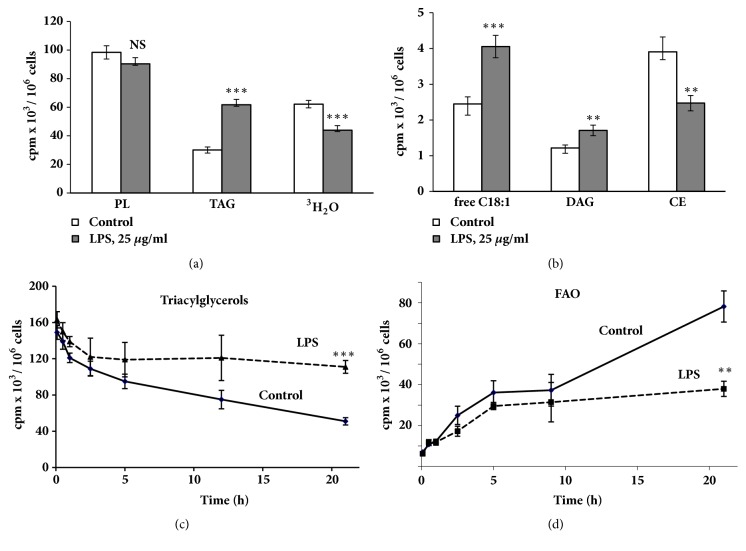
Incubation of FUBEC with LPS changes the distribution of [^3^H]-C18:1 between lipid classes**. (a, b)** Effect of LPS on the metabolism of different lipid classes. PL: phospholipids, TAG: triacylglycerols, DAG: diacylglycerols, CE: cholesterol esters. ((**c), (d)**) Time-course of the effect of LPS on [^3^H]-TAG level and on [^3^H]-C18:1 oxidation. Cells were preloaded with [^3^H]-С18:1 for 1 h and then rinsed and incubated with LPS (25 *μ*g/ml, 21 h). At the end of the incubation, cells were harvested and total lipid extraction and TLC were performed. n=6 independent experiments. *∗∗* p< 0.01; *∗∗∗* p<0.001 versus control.

LPS caused an increase of radioactivity in TAG, diacylglycerol, and free oleic acid, as well as a reduction of radioactivity in CE (Figures 2(a) and 2(b)). Evaluation of FAO showed that LPS caused a decrease in [^3^H]-oleic acid oxidation (Figure 2(a)). These results are in a good agreement with data obtained by other authors. Impaired FAO due to LPS was observed in macrophages [3, 4], dendritic cells [19], and AC16 cells [11], as well as in the liver, kidney, and heart following systemic administration of LPS [7–9].

Because LPS was added after the removal of nonincorporated [^3^H]-oleate from the extracellular medium, the increase of radioactivity in TAG could be caused by the redistribution of the label between lipid classes. The labelling of PLs was unchanged whereas FAO was reduced and the content of [3H]-oleic acid was significantly elevated (Figures 2(a) and 2(b)). These data allow us to suggest that the reduction of FAO is the main reason for the increased amount of TAG in LPS-stimulated cells.

This suggestion was confirmed by a time-course analysis of the effect of LPS. During the first 5 hours of incubation, both control and stimulated cells consumed TAG and oxidized fatty acids at a similar rate. Then, control cells continued TAG consumption and oxidation of fatty acids at a nearly constant rate (Figures 2(c) and 2(d)), whereas in the presence of LPS these processes were significantly slowed. After 21 h of incubation, the differences became twofold (Figures 2(c) and 2(d)).

### 3.3. Effect of LPS on OCR, Mitochondrial Membrane Potential, and Production of ATP

**Figure 3:**
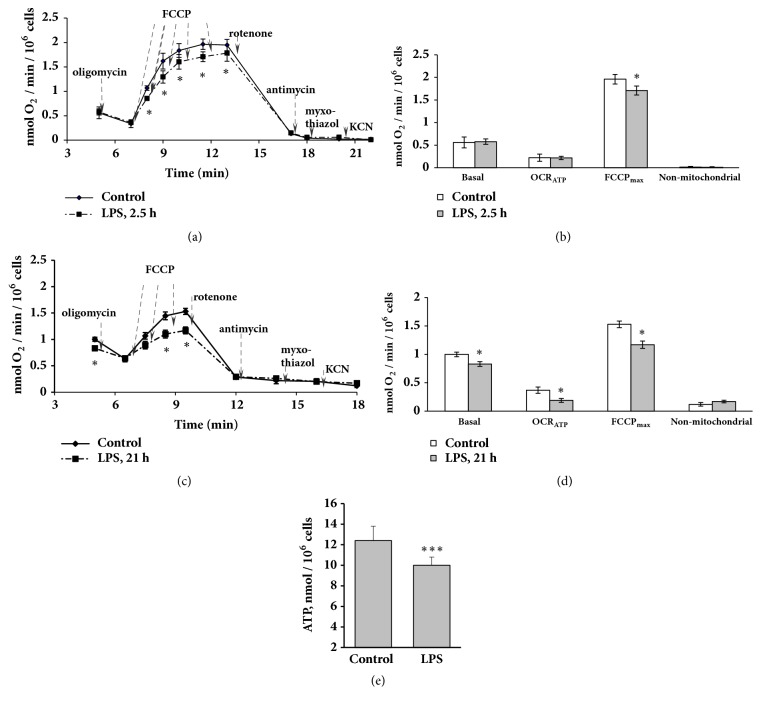
Effect of LPS on the oxygen consumption rate under different respiratory conditions and on ATP synthesis. OCR by FUBEC incubated with or without LPS (25 *μ*g/ml) for 2.5 h** (a, b) **and 21 h** (c, d) **was analyzed. ((a), (c)) Respirometry experiments; ((b), (d)) quantification of data. Arrows indicate the addition of drugs. n=6 independent experiments. (**e**) ATP production by FUBEC, incubated with or without LPS (25 *μ*g/ml, 21 h), n=22. *∗* р<0.05; *∗∗∗* р<0.001 versus control.

Based on the results that LPS inhibits FAO, next we tested whether LPS affected mitochondrial function in FUBEC. OCR measurements revealed that application of LPS at a dose that declined FAO (25 *μ*g/ml) already after 2.5 h of incubation led to the reduction of OCR_max_ in the presence of the uncoupler FCCP (Figures 3(a) and 3(b)) indicating decreased mitochondrial effectiveness in the presence of LPS. After 21 h of incubation with LPS, besides the decline of the uncoupler effect, a significant decrease in basal OCR (LPS versus control) was observed (Figures 3(c) and 3(d)). OCR in the presence of oligomycin (ATP-synthase inhibitor) did not differ in control and LPS-stimulated cells. Thus, the oxygen consumption required for ATP synthesis (OCR_ATP_) in LPS-stimulated cells was lower than that in control cells whereas H^+^-leaking remained unchanged. It should be noted that the addition of oligomycin in a FCCP background did not lower OCR (data are not shown) indicating the absence of other effects of oligomycin on respiration besides ATP-synthase inhibition. Nonmitochondrial respiration was the same for control and LPS-stimulated cells, independent of the duration of incubation (Figures 3(a)–3(d)).

As shown in Figure 3(e), a 21 h incubation with LPS resulted in a small in magnitude but statistically significant decrease of the ATP level, indicating an energy deficiency in FUBEC after prolonged LPS challenge.

**Figure 4:**
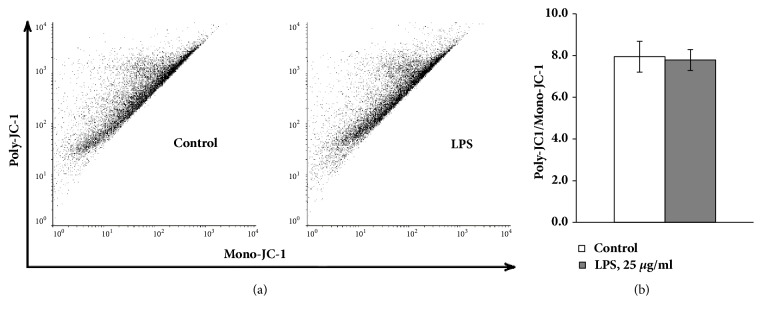
LPS does not change MPP in FUBEC. Cells were incubated with 25 *μ*g/ml LPS for 21 h followed by staining with JC-1 and red/green image density measurements. The green fluorescence density indicated JC-1 monomers while the red fluorescence density JC-1-polymers. n=4 independent experiments.

MPP is another indicator of mitochondrial bioenergetics function. FUBEC staining with JC-1 revealed that LPS treatment did not induce any detectable changes of MPP (Figure 4) indicating preservation of the mitochondrial integrity in the presence of LPS.

### 3.4. Selection of the MitoQ Concentration with the Use of a Cytotoxicity Test and OCR Measurement

Taking into account the fact that mitochondria are one of the main sources of ROS and that LPS in FUBEC targets mitochondrial function, the mitochondrial-targeted antioxidant MitoQ was chosen for use in the following experiments. Even if its protective effect against different pathological stimuli was demonstrated both* in vivo* [26, 27] and* in vitro* [24, 28], MitoQ can possess prooxidant properties stimulating superoxide and H_2_O_2_ production [35, 36] and decrease OCR_ATP_, OCP_max_, and MMP [35, 37]. Additionally, it should be mentioned that the concentration of MitoQ used in* in vitro* experiments varies greatly in the literature—from 1 nM [36] to 300-500 nM [35, 38] or even 1 *μ*M [24, 38, 39]. In this context, it was necessary to choose concentration of MitoQ that was suitable for our cellular model.

**Figure 5:**
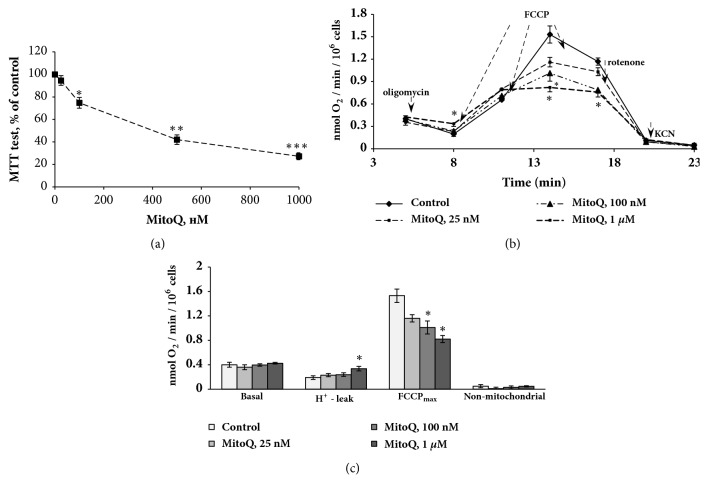
Evaluation of MitoQ toxicity on FUBEC. (**a**) MTT-test, 21 h incubation with MitoQ. (**b, c**) Oxygen consumption rate (3 h incubation with MitoQ). (b) Respirometry experiments; (c) quantification of data. n=4 independent experiments; p< 0.05; *∗∗* p< 0.01; *∗∗∗* p<0.001 versus control.

The toxicity of MitoQ in a concentration range from 25 nM to 1 *μ*M was evaluated by the MTT-test. The results showed that MitoQ had a dose-dependent toxic effect in FUBEC—1 *μ*M significantly decreased the MTT-test indexes whereas 25 nm had practically no effect (Figure 5(a)). Analysis of OCR by FUBEC that were incubated for 3 h with MitoQ revealed that 1 *μ*M increased H^+^ leak and decreased OCR_max_ in the presence of FCCP, indicating a detrimental effect of this dose on mitochondrial function (Figures 5(b) and 5(c)). MitoQ at a dose of 100 nM caused decrease of OCR_max_, whereas 25 nM had only a tendency to decrease it, indicating that MitoQ at doses higher than 25 nM inhibited the ETC. Based on these data, the following experiments were performed with 25 nM MitoQ.

### 3.5. MitoQ Did Not Prevent the LPS-Induced Decline of *OCR*_*max*_ but Decreased Basal and LPS-Stimulated ROS Production

To test whether MitoQ was able to influence the LPS-induced decline of OCR, we preincubated cells with 25 nM MitoQ for 2 h followed by a 21 h incubation with LPS. The measurement of OCR revealed that MitoQ did not prevent the LPS-induced decrease of OCR_max_, and even there was a trend toward an additive effect for the two drugs (data not shown).

**Figure 6:**
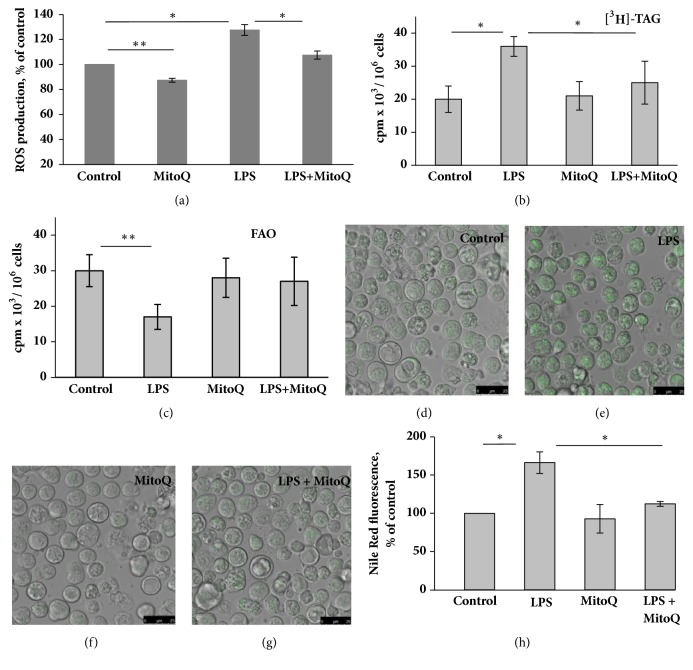
MitoQ decreases basal and LPS-stimulated ROS production** (a)** and prevented LPS-induced TAG accumulation** (b)**, decline of FAO** (c),** and LD biogenesis** (d-h)**.** (a) C**ells were pretreated with MitoQ for 2 h and then exposed to 25 *μ*g/ml LPS for 1 h. The fluorescent dye, DCF-DA, was added to the incubation medium at a final concentration of 10 *μ*M, 20 min before the end of incubation with LPS, n = 6. ((**b)-(h)) **Cells were pretreated for 2 h with MitoQ (25 nM) and then incubated for 21 h with 25 *μ*g/ml LPS. ((**b), (c))** Cells were preloaded with [^3^H]-С18:1 during 1 h, n=6 independent experiments; ((**d)-(h))** Cells were stained with Nile Red, n=4 independent experiments. *∗* p< 0.05; *∗∗* p< 0.01.

To analyze the antioxidant capacity of MitoQ, FUBEC were preincubated for 2 h with 25 nM MitoQ prior to a 1 h incubation with LPS. Antioxidant treatment resulted in the suppression of both basal and LPS-stimulated ROS production (Figure 6(a)). *α*-Tocopherol, a nonmitochondrial antioxidant, at concentrations 10 and 50 *μ*M, did not reduce LPS-stimulated ROS production, and, by itself, demonstrated rather weak prooxidant properties (data not shown).

### 3.6. MitoQ Prevented LPS-Induced FAO Decline, TAG Accumulation, and LD Formation

To examine the involvement of mROS in LPS-induced lipid metabolic shift, we analyzed the effect of MitoQ on the LPS-stimulated changes of lipid metabolism in cells preincubated with [^3^H]-oleate. While LPS led to a significant increase in TAG radioactivity and a decrease in the level of FAO, MitoQ significantly suppressed both effects (Figures 6(b) and 6(c)). *α*-Tocopherol (10 and 50 *μ*M ), which did not possess antioxidant properties in FUBEC, also had no effect on LPS-induced lipid metabolism alterations (data not shown). Nile Red staining of FUBEC preincubated with 25 nM MitoQ for 2 h revealed that MitoQ* per se* did not change the number or size of LD but almost completely eliminated the effect of LPS on LD biogenesis (Figures 6(d)–6(h)).

## 4. Discussion

This study was designed to clarify whether mROS contribute to the LPS-induced shift of lipid metabolism in epithelial cells. First, we obtained data that accumulation of TAG in FUBEC could be attributed to an LPS-induced decline of mitochondrial FAO leading to subsequent TAG retention. This suggestion is based on the LPS-induced intracellular accumulation of nonoxidized fatty acids and inhibition of FAO and on a time-course of the effect of LPS on the rate of FAO and TAG breakdown.

We further demonstrated that LPS treatment actually increased cellular ROS production and led to the impairment of mitochondrial function, which was revealed by reduced OCR_max_ and OCR_ATP_ as well as by the decrease of ATP production. The data suggest that LPS affects the respiratory chain, decreasing oxidative phosphorylation. However, LPS treatment did not appear to dramatically change the respiratory function of FUBEC mitochondria, since the effect of LPS on OCR was not accompanied by an increase of H^+^ leak or a decrease of MMP, indicating the preservation of mitochondrial integrity. Next, we determined the appropriate concentration of MitoQ by testing its toxicity with a MTT-test and OCR evaluation. The data obtained indicated that FUBEC were highly sensitive to the toxic effect of MitoQ and that the dose applied in our study (25 nM) was significantly less than was generally used in most* in vitro* works [28, 35, 38, 39]. MitoQ did not prevent the LPS-induced decrease of OCR_max_ but reduced the effect of LPS on ROS, indicating the existence of different targets and mechanisms of its action within mitochondria. The study of LPS-induced lipid metabolic changes in the presence of MitoQ revealed that a mitochondrial-targeted antioxidant restored the LPS-induced decline of FAO and prevented accumulation of TAG and LD biogenesis.

Since the ROS measurement was performed with the use of DCF-DA, the data obtained do not allow for differentiation between ROS of mitochondrial and nonmitochondrial origin. However it seems to be more important that the LPS-stimulated production of ROS and lipid metabolic shift were prevented by a mitochondrial-targeted antioxidant, but not a nonmitochondrial one (*α*-tocopherol). Given the multiplicity of ROS-producing sources in mitochondria, various mechanisms of LPS-induced ROS generation inside mitochondria could be proposed. For example, LPS was shown to reduce complex I activity, leading to increased production of superoxide and H_2_O_2_ [40]. Complex I is one of the main sources of mitochondrial ROS [22, 41], and its dysfunction can be a trigger for inflammatory responses. The intrinsic mechanism of LPS-induced generation of mitochondrial ROS coupled with TRAF6-mediated mitochondrial complex I impairment has been revealed in macrophages [42]. However, whether such a mechanism exists in other cell types is unclear.

The potential mechanisms of mROS-mediated inhibition of FAO and TAG retention in response to LPS could not be completely understood in the frame of the present study. The enzymatic activity of carnitine palmitoyltransferase I (CPT-1), the rate-limiting enzyme for the *β*-oxidation of fatty acids, can be significant and rapidly (within 30 min) downregulated by ROS [43]. CPT-1 may be subjected to posttranslational modifications by ROS-mediated lipid peroxidation products resulting in a sharp decrease of its activity [44]. However, our data on a time-course analysis of the LPS effect indicate that statistically significant inhibition of FAO was observed much later (at least, after 9 h of incubation) than the LPS effects on ROS production (1 h) or influences on OCR (2.5 h). These data suggest that the effect of LPS is rather related to mROS-triggered control of expression of proteins which contributed to FAO regulation or TAG breakdown. Unfortunately, we failed to demonstrate convincingly the influence of LPS and MitoQ on CPT-1 expression by immunoblotting due to insufficient specificity of commercial antibodies for FUBEC. However, both* in vivo * and* in vitro,* it was shown that LPS reduced the expression of CPT-1 as well as transcription factors PPAR*α* and PGC-1*α* [3, 7, 8, 11, 12]. Reduced PPAR*γ* expression was shown to mediate TAG accumulation caused by antimycin-induced mitochondrial dysfunction [13]. The high level of mROS has been reported to promote activation of the redox-sensitive transcriptional factor NF-kB, which plays a central role in LPS signaling, in cells challenged with LPS [45, 46]. Mitochondrial-targeted antioxidants were shown to reduce the LPS-triggered inflammatory response by regulating the NF-*κ*B pathway in HUVEC [47], microglia cells [31], and C6 glioma cells [48]. Taking into account the extremely broad spectrum of ROS signaling, all of the above-mentioned mechanisms of mitochondrial FAO control could be targeted by mROS.

Regardless of the mechanism, our data demonstrate, for the first time, the key role of mROS in inhibition of mitochondrial FAO, TAG retention, and LD biogenesis under the influence of a bacterial pathogen. Our results are in a good agreement with the data of Boren and Brindle [24] who demonstrated a key role of mROS in the decline of FAO in etoposide-induced apoptosis of murine lymphoma cells. It is important to note that the mucosal surface of the amphibian urinary bladder is commonly exposed to Gram-negative bacteria and that isolated uroepithelial cells demonstrate a relatively high tolerance to LPS. In contrast to many other cell types, LPS, at least at the dose applied in this study, does not induce apoptosis in isolated FUBEC [34]. In this context, generation of mROS in response to the LPS action on mitochondria and activation of downstream signaling pathways that mediate FAO decline and TAG retention could be rather considered as a mechanism of metabolic downregulation, representing an adaptive cellular response to bacterial pathogens.

## Data Availability

The data used to support the findings of this study are available from the corresponding author upon request.

## Abbreviations

ARS: Amphibian Ringer solution
CE: Cholesterol esters
ETC: Electron transport chain
FAO: Fatty acid oxidation
FUBEC: Frog urinary bladder epithelial cells
LD: Lipid droplets
LPS: Lipopolysaccharide
MPP: Mitochondrial membrane potential
mROS: Mitochondrial reactive oxygen species
OCR: Oxygen consumption rate
ROS: Reactive oxygen species
TAG: Triacylglycerol
TLC: Thin-layer chromatography.
